# Diagnosis, treatment characteristics, and survival of women with breast cancer aged 65 and above: a hospital-based retrospective study

**DOI:** 10.1186/1472-6874-13-34

**Published:** 2013-08-28

**Authors:** Mehtap Kartal, Sabahat Tezcan, Tulay Canda

**Affiliations:** 1 Family Medicine Department of Dokuz Eylul University, Inciralti, Izmir, 35340, Turkey; 2 Public Health Department of Hacettepe University, Ankara, Turkey; 3 Pathology Department of Dokuz Eylul University, Inciralti, Izmir, 35340, Turkey

**Keywords:** Aged, Women, Breast neoplasms, Survival

## Abstract

**Background:**

Breast cancer incidence in women increases with age, while survival rates decrease. Studies interpret this result as meaning higher comorbidity, diagnosis at later stages of the disease, and less effective treatment in the elderly. The aim of this study is to evaluate the diagnosis and treatment characteristics of breast cancer and their effect on the survival of women aged 65 and above.

**Methods:**

The data within the files of 1064 women with breast cancer, who were followed-up in Dokuz Eylul University Medical Faculty Hospital between 2000 and 2006, were reviewed retrospectively. The survival probabilities at years 1 and 5 were calculated by life table analysis. The Kaplan-Meier test was used for calculating mean survival time, and the differences between groups were evaluated by log-rank test. The backward elimination method was used for multivariate analysis, and a −2 log-likelihood ratio was used for comparison of different models.

**Results:**

Of the patients, 25.3% were aged 65 and above at the time of the diagnosis. Patients in this group had more comorbidities and were more likely to be diagnosed at advanced stages than younger patients. Additionally, they had lower rates of surgical treatment, chemotherapy or radiotherapy. One and 5-year survival probabilities among age groups were 96.1% and 84.5%, respectively, for <65 years, 93.5% and 84.8%, respectively, for 65–69, 98.7% and 84.0%, respectively, for 70–74, and 85.5% and 59.6%, respectively, for 75 years and above. In the multivariate model, age, clinical stage, and comorbidity were found to be negatively associated with the survival rate.

**Conclusions:**

The survival of women with breast cancer aged 65 and above was affected negatively by age at diagnosis, clinical stage, and the presence of comorbidity. Early diagnosis also is very important for elderly women. Additionally, because of higher comorbidity, their evaluation and treatment should be planned by an interdisciplinary team.

## Background

It is known that breast cancer incidence is rather low before the age of 30 (25 in 100,000) and demonstrates a linear increase through the age of 80, where it reaches a plateau (around 500 in 100,000). Age is the most important risk factor in breast cancer occurrence after gender. It is observed that the relative risk for breast cancer increases by 5.8 times in women aged 65 and above compared with women below age 65 [1]. Projections by the United Nations have shown that breast cancer incidence is increasing rather rapidly among women aged 50 and above [2]. In Turkey, the prevalence of breast cancer based on records of women varies between 21.4 and 45.6 per 100,000, placing this disease at the top in terms of frequency of occurrence [3]. It was seen that among 11,208 women with breast cancer registered to 13 breast centers in eight provinces, 40.7% were between 51 and 70 years of age and 8.2% were aged 70 and above [4].

It is known that the survival of women with breast cancer diagnosed at later ages is worse than that of younger women. In relation to this, cardiovascular diseases, pre-existing cancer, diabetes mellitus and dementia are mentioned among those diseases that most frequently accompany breast cancer in the elderly. It has also been pointed out that these diseases increase the risk of death [5-7]. The reasons why survival is lower in the elderly while breast cancer in this age group displays less aggressive tumor characteristics compared with the young have been examined. Among the reported reasons are that the diagnosis occurs in later stages and that the elderly receive less surgical treatment, are insufficiently treated, and receive less radiotherapy following breast-conserving surgery and chemotherapy [7-11]. The fact that patients from older age groups do not participate in many randomized controlled studies that assess the effectiveness of chemotherapies has meant that the most appropriate treatment for the elderly is still being discussed [12].

When the survival probabilities of women were assessed by age, it was seen that all of the women aged 65 years and above who were diagnosed in stage 0 and 1 were alive at the end of 5 years, while their survival was between 83.9% and 87.8% in stage 2, 41.5% and 57.8% in stage 3, and 14.8% and 20.3% in stage 4 [13]. Among the factors that influence the survival of patients with breast cancer, histological grade and the presence of lymphovascular invasion (LVI), particularly for those with negative lymph node, are significant apart from clinical staging [14-17].

In light of previous studies, the aim of this study was to determine certain diagnostic and treatment characteristics of women with breast cancer aged 65 and above, and compare their overall survival time, survival probabilities, and factors that might influence their survival with women younger than 65 years of age.

## Methods

The study included 1064 female patients who were followed up with a breast cancer diagnosis and/or treatment in Dokuz Eylul University Medical Faculty Hospital between January 1^st^, 2000 and December 31^st^, 2006. The data on the patients were obtained by examining the patient files, hospital information systems and pathological reports retrospectively. The study was completed after the final monitoring status of the patients determined on December 31^st^, 2011. Approval was obtained from Dokuz Eylul University Medical Faculty Hospital Ethics Committee.

Cox proportional hazards models were used to evaluate the associations of age, comorbidity, clinical stage, histological grade, lymphovascular invasion, recurrence and metastasis in the follow-up period with mortality. Survival status was censored at the date of last in-person contact noted patient files, hospital information systems or December 31^st^, 2011. The duration from diagnosis to death was accepted as overall survival time. The ages of the patients were given as <65, 65–69, 70–74, ≥75 years. Diseases included in comorbid conditions were cardiovascular diseases, pre-existing cancer, diabetes mellitus as they are the diseases that most frequently accompany breast cancer in the elderly.

### Statistical analysis

Categorical variables were presented with frequency and percentage distributions, while continuous variables were given with mean and standard deviation values.

The 5-year survival probabilities were calculated by life table analysis. The mean survival times were calculated with the Kaplan-Meier test, and the differences between groups were evaluated by the log-rank test. The prognostic values of all related variables were first assessed in univariate analyses. Then the variables that had significant association with the outcome were included in a multivariate Cox proportional hazard model using the backward elimination method, with −2 log-likelihood ratio values taken into consideration for comparison of different models. The proportional hazards assumption was checked with a log minus log plot for each variable in the model.

The data were analyzed with the SPSS 15.0 program. All reported p values are two-tailed and p<0.05 was accepted as statistically significant.

## Results

The mean follow up period was 74.6±39.8 months with a median of 77 months. Certain characteristics of the women included in the study are given in Table 1. Ages of the patients at the time of diagnosis ranges between 24 and 90 years, and 25.3% were aged ≥65 years. The mean age of this subgroup was 71.9±5.4 years at the time of the diagnosis.

**Table 1:** Descriptive characteristics of women with breast cancer

		**n**	**%**
Age at the time of diagnosis (n=1036)	<65	774	74.7
	65-69	102	9.8
	70-74	92	8.9
	≥75	68	6.6
Year of diagnosis (n=1036)	2000	147	14.2
	2001	137	13.2
	2002	131	12.6
	2003	152	14.7
	2004	143	13.8
	2005	164	15.8
	2006	162	15.6
Comorbidity (n=690)	No	557	80.7
	Yes	133	19.3
Stage (n=912)	1	208	22.8
	2	415	45.5
	3	197	21.6
	4	92	10.1
Histology (n=1012)	Invasive ductal ca	473	46.7
	Invasive lobular ca	220	21.7
	Mixed	182	18.0
	Others	137	13.5
Histologic grade (n=745)	1	119	16.0
	2	338	45.3
	3	288	38.7
Lymphovascular invasion (n=849)	No	174	20.5
	Yes	675	79.5
Surgery (n=1010)	No	52	5.1
	Yes	958	94.9
Chemotherapy (n=824)	No	187	22.7
	Yes	637	77.3
Radiotherapy (n=794)	No	28	3.5
	Yes	766	96.5
Hormonotherapy (n=703)	No	113	16.1
	Yes	590	83.9
Recurrence (n=545)	No	490	89.9
	Yes	55	10.1
Metastases (n=608)	No	415	68.3
	Yes	193	31.7

The distribution of some characteristics of the patients in terms of age is summarized in Table 2. Women aged >65 years had more comorbid diseases than younger women (p<0.001). The clinical stages in which the women were diagnosed in terms of their ages were found not to be significantly different (p=0.068), with 19.2% of those ≥75 years at clinical stage 4 at diagnosis. Patients aged <65 years received more surgical treatment (97.4%), chemotherapy (82.5%) and radiotherapy (97.6%) than those who were older.

**Table 2:** Some characteristics of women with breast cancer according to their age at the time of diagnosis

	**Age groups n (%)**				**p-value**
	**<65**	**65-69**	**70-74**	**≥75**	
Comorbidity (n=686)					<0.001
No	437 (88.1)	49 (65.3)	40 (60.6)	28 (57.1)	
Yes	59 (11.9)	26 (34.7)	26 (39.4)	21 (42.9)	
Stage (n=906)					0.068
1	167 (24.3)	15 (17.0)	17 (21.8)	9 (17.3)	
2	313 (45.5)	42 (47.7)	35 (44.9)	24 (46.2)	
3	154 (22.4)	18 (20.5)	14 (17.9)	9 (17.3)	
4	54 (7.8)	13 (14.8)	12 (15.4)	10 (19.2)	
Histologic grade (n=740)					0.034
1	84 (14.9)	11 (16.4)	12 (18.2)	12 (27.9)	
2	249 (44.1)	35 (52.3)	29 (43.9)	24 (55.8)	
3	231 (41.0)	21 (31.3)	25 (37.9)	7 (16.3)	
Lenfovascular invasion (n=845)					0.161
No	133 (20.5)	21 (28.0)	9 (12.9)	10 (19.2)	
Yes	515 (79.5)	54 (72.0)	61 (87.1)	42 (80.8)	
Surgery (n=1002)					<0.001
No	20 (2.6)	9 (9.6)	9 (10.3)	11 (17.2)	
Yes	737 (97.4)	85 (90.4)	78 (89.7)	53 (82.8)	
Chemotherapy (n=818)					<0.001
No	112 (17.5)	18 (24.0)	30 (48.4)	27 (64.3)	
Yes	527 (82.5)	57 (76.0)	32 (51.6)	15 (35.7)	
Radiotherapy (n=791)					0.010
No	15 (2.4)	4 (5.6)	5 (8.3)	4 (9.3)	
Yes	601 (97.6)	68 (94.4)	55 (91.7)	39 (90.7)	
Hormonal therapy (n=697)					0.110
No	96 (18.0)	8 (13.1)	6 (10.5)	3 (6.7)	
Yes	438 (82.0)	53 (86.9)	51 (89.5)	42 (93.3)	
Recurrence (n=543)					0.246
No	380 (90.9)	44 (84.6)	38 (86.4)	28 (96.6)	
Yes	38 (9.1)	8 (15.4)	6 (13.6)	1 (3.4)	
Metastasis (n=604)					0.755
No	324 (69.7)	38 (66.7)	30 (62.5)	23 (67.6)	
Yes	141 (30.3)	19 (33.3)	18 (37.5)	11 (32.4)	

The mean survival times for the age groups were 121.0 months for the <65 years of age group, 119.0 months for the 65–69 years of age group, 121.4 months for the 70–74 years of age group, and 85.2 months for the ≥75 years of age group (median survival time=86.0 months) (Log rank chi-square=16.288; p<0.001). The 5-year survival probabilities of patients in terms of age group were 84.5% for age <65 years, 84.8% for age 65–69 years, 84.0% for age 70–74 years, and 59.6% for age ≥75 years. The survival probabilities by age group and clinical stage are given in Table 3.

**Table 3:** Survival rates of women with breast cancer according to their age and stage at the time of diagnosis

	**Stages**	**Age groups**			
		**<65**	**65-69**	**70-74**	**≥75**
	1	98.0	91.7	94.1	75.2
5 year survival*	2	91.4	92.5	89.8	77.4
	3	72.5	81.0	72.5	37.5
	4	23.4	40.2	43.2	33.8

*Life table analysis.

**Figure 1:**
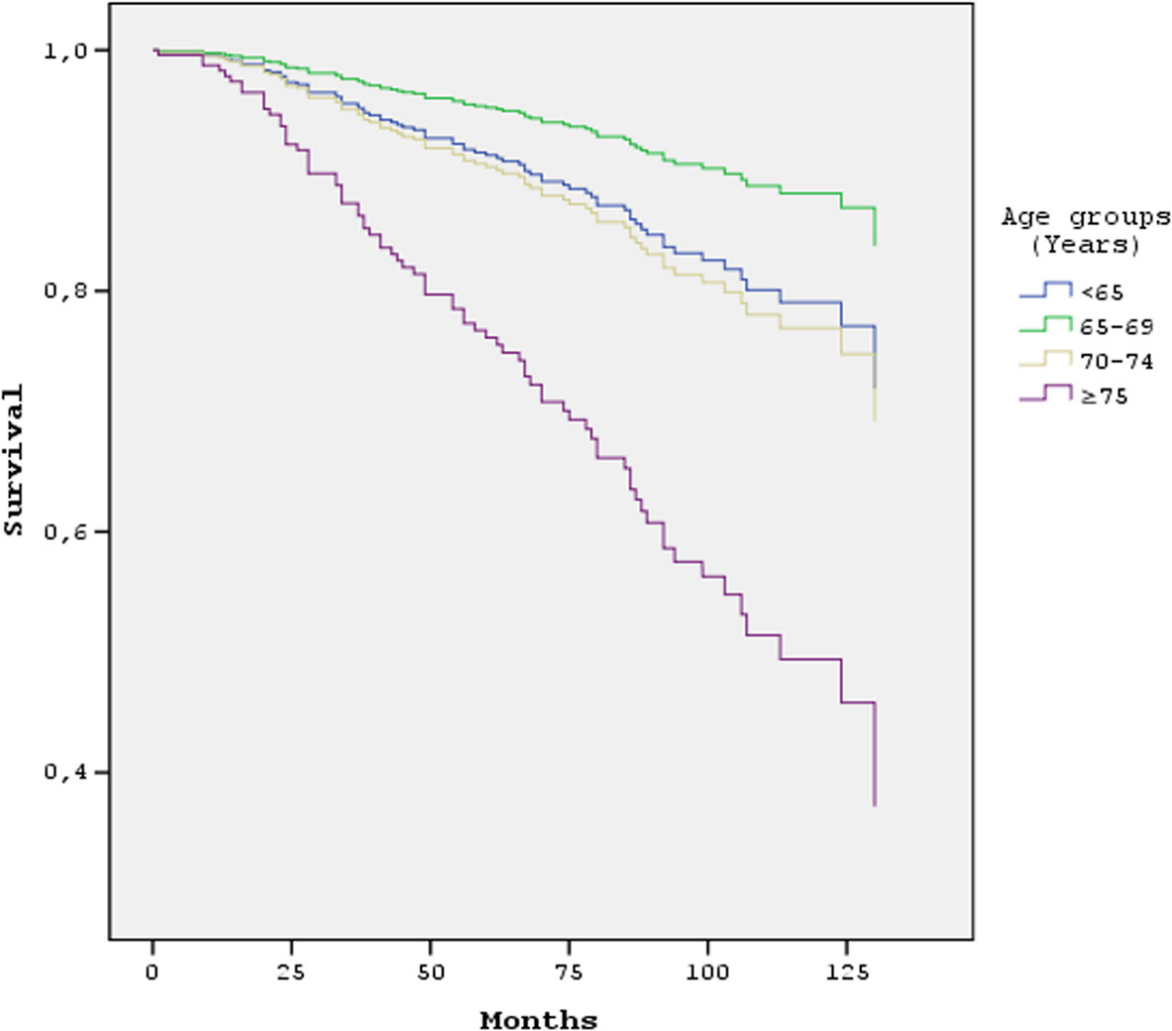
Survival curves of women with breast cancer according to their age at the time of diagnosis.

In addition to age and clinical stage, the final model included co-morbid disease, histological grade, surgical treatment, chemotherapy and radiotherapy. These additional factors are shown to influence survival and be significantly different among groups. When all these variables were corrected in relation to one another but no variables were eliminated, age, clinical stage and comorbidity was found to be statistically significant (p<0.05). The reduced model included age, clinical stage, comorbidity and histologic grade (Table 4). In the ≥75 years of age group, increasing clinical stage and histologic grade, presence of comorbidity increased the hazard ratios (HR) of the patients. Presence of comorbidity increased the HR 2.3 times independently of the age at diagnosis, clinical stage and histological grade of the disease. When the survival curves of the reduced model are evaluated, the survival probabilities of the patients aged <65 years were found to be similar to 70–75 years of age group while 65–69 years of age group was better than both of them. And women over 75 years of age distinctly differ from younger women in all age groups which may be interpreted as age of 75 may be a breaking point for survival (Figure 1).

**Table 4:** Cox-regression models showing factors affecting the survival of women with breast cancer

	**Full model**				**Reduced model**			
	**HR**	**%95 CI**		**p**	**HR**	**%95 CI**		**p**
Age (years)								
<65*				**0.047**				**0.025**
65-69	0.6	0.2	1.5	0.252	0.5	0.2	1.4	0.189
70-74	1.2	0.5	2.5	0.710	1.1	0.5	2.4	0.773
≥75	3.6	1.2	11.0	0.023	3.0	1.3	6.8	0.009
Comorbidity								
No*								
Yes	2.3	1.3	4.1	**0.003**	2.3	1.4	4.0	**0.002**
Stage								
1*				**<0.001**				**<0.001**
2	2.0	0.8	5.0	0.149	2.1	0.9	4.9	0.072
3	6.4	2.4	16.7	<0.001	7.1	3.0	16.4	<0.001
4	21.5	6.5	70.6	<0.001	22.5	8.7	58.4	<0.001
Histologic grade								
1*				0.085				0.075
2	1.8	0.8	4.2	0.152	1.8	0.8	4.2	0.149
3	2.5	1.1	5.8	0.033	2.5	1.1	5.8	0.030
Surgical treatment								
Yes*								
No	1.1	0.3	3.3	0.899				
Chemotherapy								
Yes*								
No	1.2	0.5	3.1	0.680				
Radiotherapy								
Yes*								
No	1.2	0.3	3.9	0.819				
−2 log likelihood ratio	788.363				788.659			

*Indicates reference category.

*HR* Hazard ratio (ß).

*CI* Confidence interval.

## Discussion

The number of women with breast cancer increases with increase in life span and increase in elderly population, which in turn results in a higher increase in the number of elderly women with cancer. Breast cancer particularly affects elderly women in developed countries [9]. A multicentre study including 11,208 breast cancer patients from June 2005 until February 2008 showed that 8.2% were aged above 70 years, while in this study, patients aged above 65 years constituted 25.0% of all the patients [4]. The mean age of our patients above 65 years was 71.9 years, while another single-centered study including breast cancer patients aged above 65 years found the mean age of an equivalent group to be 72.7 years [18].

Co-morbid diseases accompanying breast cancer occur more frequently in elderly patients. It is known that in the elderly without any comorbid diseases, the disease displays a relatively good prognosis, whereas in those with comorbid diseases, it has rather a poor one. It was found in one study that comorbid disease increases the hazard ratio by 1.3 times independent of age, stage and treatment [6,8]. In our study, 34.2-44.4% of women aged ≥65 years have at least one disease accompanying breast cancer, while only 11.9% of those aged ≤65 years have at least one co-morbid disease. In the multivariate analysis, co-morbid disease was found to increase the hazard ratio by 2.3 times.

Impairments in organ functions, which occur as a result of increased age as well as co-morbid diseases in the elderly, might prevent the formulation of a management plan so that optimum treatment can be achieved. As a consequence, elderly patients cannot be treated in an aggressive way though they are diagnosed at later stages than younger patients [9,19,20]. When assessed from the viewpoint of surgical treatment, it has been reported that a more limited surgical treatment is administered in the elderly and that this situation is much more apparent in patients aged ≥80 years [6]. It has been shown that breast-conserving surgery is performed less often and that this situation continues even after recovery from the co-morbid disease is achieved [6-8]. The situation is similar with radiotherapy and chemotherapy. While in various studies patients aged ≥70 and ≥80 years are emphasized, the consensus is that the patients in this age group receive less chemotherapy and radiotherapy but more hormonal therapy [6,7,19,20].

Even though it is known that patients are diagnosed later with more advanced age, it has been shown that this situation is also true for patients over 70. While patients diagnosed at stage 4 constitute 3.7% of people aged <65 years, they constitute 8.5% of the age group 80–84 [20,21]. Similarly, we have also found in our study that women aged ≥75 years could not benefit from improved staging methods and therapies. They were diagnosed at later stages which can be due to the gab in the screening programs or the ignorance of the disease and rejection of its hard treatment process by the elderly as these can easily affect their quality of life. They received less surgical treatment, chemotherapy and radiotherapy compared with patients in other age groups. This result might indicate that clinicians are caught between the short-term damage of special interventions in breast cancer and the death of patients due to other factors. Moreover, some studies indicate that breast cancer progresses less aggressively in the elderly and thus the usual treatment approaches are not required. However, the knowledge on this issue is limited and further research is needed [9,18].

In the EUROCARE-3 study, the survival probabilities of women at 5 years were 76% for ages 65–74 years and 69% for ages above 75 years [22]. In SEER records, on the other hand, survival at 5 years was 89.2% for women aged ≤65 years, 90.4% for ages 65–74 and 86.8% for ages above 75 years [23]. In a study conducted in Turkey, survival at 5 years for women aged ≥65 years was 62%. In the same study, stage and lymph node involvement were reported to be the most important factors affecting survival [18]. In our study, it was observed that survival probabilities at 5 years decrease with increased age, to 59.6% for ages above 75 years. When age and clinical stage, which affect the survival rate of patients with breast cancer, were evaluated together, the age of 75 appears to be a breaking point with markedly decreased survival rate.

In studies that assess the effect of the increasing inadequacies and deficiencies in treatment due to age on patient survival, different results were obtained; nevertheless, it has been shown that radiotherapy increased the overall and disease-free survival while this influence could not be confirmed for surgical treatment, radiotherapy and chemotherapy [7,10]. In the modeling made for this study, it can also be seen that the presence of co-morbid disease in addition to age and clinical stage influenced total survival significantly, whereas surgical treatment, radiotherapy and chemotherapy did not have any significant influence on overall survival.

The most important limitation of our study is that the data were collected retrospectively from existing patient files, which brings the limitations of recorded patient information, including certain shortcomings and possible mistakes.

## Conclusions

In summary, women aged ≥65 years were diagnosed with later stages of breast cancer. The co-morbid diseases of these patients might limit physician choices of treatment. Increased age, clinical stage and co-morbid diseases had a negative impact on survival. The early diagnosis of breast cancer is as important for the elderly as for the young and it will be essential to re-evaluate the upper age limit defined in national breast cancer screening programs. Furthermore, planning the post-diagnosis assessment and treatment of the functional losses and co-morbid diseases occurring with aging with an interdisciplinary team are important for the survival of patients.

## Competing interests

The authors declare that they have no competing interests.

## Authors’ contributions

MK participated in the design and data collection of the study, performed the statistical analysis, and involved in drafting the manuscript. ST participated in the design of the study and involved in drafting and revising the manuscript. TC helped to draft the manuscript. The manuscript has been seen and approved by all authors.

## Pre-publication history

The pre-publication history for this paper can be accessed here:

http://www.biomedcentral.com/1472-6874/13/34/prepub
